# Deep learning-based automation for segmentation and biometric measurement of the gestational sac in ultrasound images

**DOI:** 10.3389/fped.2024.1453302

**Published:** 2024-12-18

**Authors:** Hafiz Muhammad Danish, Zobia Suhail, Faiza Farooq

**Affiliations:** ^1^ Department of Computer Science, University of the Punjab, Lahore, Pakistan; ^2^ Department of Radiology, University of Lahore Teaching Hospital, Lahore, Pakistan

**Keywords:** gestational sac, automatic segmentation, fetal biometry, early pregnancy, ultrasound images, deep learning

## Abstract

**Introduction:**

Monitoring the morphological features of the gestational sac (GS) and measuring the mean sac diameter (MSD) during early pregnancy are essential for predicting spontaneous miscarriage and estimating gestational age (GA). However, the manual process is labor-intensive and highly dependent on the sonographer's expertise. This study aims to develop an automated pipeline to assist sonographers in accurately segmenting the GS and estimating GA.

**Methods:**

A novel dataset of 500 ultrasound (US) scans, taken between 4 and 10 weeks of gestation, was prepared. Four widely used fully convolutional neural networks: UNet, UNet++, DeepLabV3, and ResUNet were modified by replacing their encoders with a pre-trained ResNet50. These models were trained and evaluated using 5-fold cross-validation to identify the optimal approach for GS segmentation. Subsequently, novel biometry was introduced to assess GA automatically, and the system's performance was compared with that of sonographers.

**Results:**

The ResUNet model demonstrated the best performance among the tested architectures, achieving mean Intersection over Union (IoU), Dice, Recall, and Precision values of 0.946, 0.978, 0.987, and 0.958, respectively. The discrepancy between the GA estimations provided by the sonographers and the biometry algorithm was measured at a Mean Absolute Error (MAE) of 0.07 weeks.

**Conclusion:**

The proposed pipeline offers a precise and reliable alternative to conventional manual measurements for GS segmentation and GA estimation. Furthermore, its potential extends to segmenting and measuring other fetal components in future studies.

## 1 Introduction

Early miscarriage, characterized by the sudden termination of a pregnancy in the initial trimester, is a common complication that impacts approximately 20% of pregnancies (1). It is an adverse pregnancy outcome that can occur despite the detection of embryonic cardiac activity, with a reported incidence ranging from 5.2% to 10.4% (2). The significant factors associated with higher rates of miscarriage are chromosomal and hormonal imbalances, infections, uncontrolled hypertension, and diabetes, consumption of alcohol and cocaine, and recurrent miscarriage history (1). Common indicators of miscarriage include abdominal cramping, vaginal bleeding, and the passing of tissues from the vagina. This distressing event profoundly impacts the mother’s mental, psychological, social, and emotional well-being, particularly for individuals experiencing recurrent miscarriages (3).

Obstetricians conduct a thorough assessment of the gestational sac’s (GS) morphological characteristics to identify signs of potential miscarriage (4). An abnormally small or large GS size compared to the expected size for the gestational week (GW) may indicate an impending miscarriage (5). At the same time, a GS with irregular or distorted contours could signal potential abnormalities or developmental issues. A heterogeneous appearance of the GS, characterized by areas of different textures, may also be associated with abnormal pregnancies (2, 6). The updated diagnostic guidelines for miscarriage by Condous et al. (7) underscore that the absence of a yolk sac (YS) alongside the mean gestational sac diameter (MSD) exceeding 25 mm or a GW greater than 7.2 weeks is indicative of an empty sac miscarriage. Therefore, accurate segmentation of the GS and precise estimation of the gestational age (GA) are clinically essential for detailed morphological analysis and reliable prediction of miscarriage risk, aiding in early intervention and patient management (8).

In clinical practice, skilled sonographers often rely on manual boundary tracing to segment the GS for morphological analysis, followed by manual measurements of the maximum length (DM) and short diameter (Dm) to estimate GA. This process depends heavily on the sonographers’ visual expertise to accurately interpret ultrasound (US) images (9). Additionally, this approach can be time-intensive and challenging, especially when analyzing a large volume of clinical images, which may affect diagnostic accuracy, consistency, and efficiency (10, 11). Existing studies (9, 12, 13) have developed approaches for GS segmentation and GA estimation, but these methods often depend on manual identification of the region of interest (ROI) and are typically limited to transvaginal sonography (TVS) scans of normal pregnancies. This reliance on manual ROI selection and restriction to specific scan types and pregnancy conditions can limit their applicability in diverse clinical settings.

The primary aim of this study was to develop an advanced pipeline with a higher degree of automation to enhance the accuracy and consistency of GS segmentation and GA estimation across diverse clinical scenarios, offering clinicians reliable support in diagnostic decision-making. This involved creating a diverse dataset comprising both normal and abnormal cases captured through TVS and transabdominal sonography (TAS), fine-tuning state-of-the-art deep learning models such as UNet, UNet++, DeepLabV3, and ResUNet, and rigorously evaluating their performance using multiple loss functions including Dice Loss (DL), Jaccard Loss (JL), and Binary Cross-Entropy Loss (BCEL), to identify the optimal model for accurate GS segmentation. Additionally, the study sought to develop an algorithm capable of accurately measuring key GS parameters like DM and Dm to enable reliable GA estimation. By comparing the automated system’s performance with that of experienced clinicians, the study aimed to validate its clinical feasibility and potential impact on diagnostic decision-making.

## 2 Related work

The segmentation of the GS plays a pivotal role in clinical decision-making and accurate GW estimation. Over the years, numerous segmentation techniques have been developed to enhance this process, including k-means clustering, active contours, region growing, thresholding, shape priors, edge detection, and deep neural networks. For instance, Khazendar et al. (14) utilized Otsu’s method (15) to determine the optimal threshold value for extracting the GS. They fitted an ellipse to the segmented GS region to calculate the MSD. Otsu’s method effectively separates the foreground from the background by minimizing intra-class variance or, equivalently, maximizing inter-class variance. This approach is particularly advantageous when dealing with images that exhibit a bimodal histogram, characterized by two distinct peaks representing the foreground and background. However, its effectiveness diminishes in cases where the histogram is unimodal or multimodal, as the pixel intensity distribution may lack a clear delineation between the foreground and background, leading to potential misclassification and inaccurate results. Another study by Zhang et al. (16) located GS in real-time frames of 2D US by manipulating coarse to fine segmentation using the AdaBoost classifier. The model achieved an average accuracy of 90±4.0%, with an average Haussdorf distance of 9.83±7.79 pixels. Notably, the model tended to underestimate the size of the GS when the GA exceeded 7 weeks.

Regional growth and active contour techniques were employed by Ibrahim et al. (17) to segment the GS and compute its geometric characteristics for the detection of early abortion. However, it is generally noted that these methods often necessitate significant contrast and a multitude of features, making them less suitable for ultrasonic image segmentation due to the considerable interference of strong noise. Yin et al. (12) introduced a semi-automatic segmentation framework aimed at aiding physicians in conducting quantitative GS analysis and miscarriage prediction. The pre-processing stage included manual cropping of the ROI, after which a coarse segmentation was performed using the region-based Chan-Vese (CV) active contour model. Subsequently, convex polygon characteristic constraints were applied to refine the results, ensuring an accurate fit for the quasi-round sacs. They utilized a private dataset of 194 US images of GS, including TVS and TAS scans, obtained during 6–9 weeks of pregnancy. The mean Dice coefficient was 91.60%, and the Intersection over Union (IoU) was 84.20%.

Pei et al. (13) identified Attention UNet as the optimal GS segmentation model and introduced a biometry for GA measurement. Their study utilized a dataset comprising 256 patients who underwent only TVS examination between 4.6 and 9.6 weeks of pregnancy. They achieved a mean Dice of 97.40%. In another study, Liu et al. (9) employed a semi-automatic technique to segment the GS, YS, and embryo regions. Initially, the original images were cropped to isolate the relevant area of interest before passing them through the AFG-net segmentation model, which is an advanced UNet incorporating Attention fusion and Guided filter modules. Their dataset consisted of 914 TVS scans captured between 6–10 weeks of gestation. They achieved a mean Dice of 96.70%.

While previous studies have demonstrated promising results, they primarily employed semi-automatic techniques that rely on manual ROI cropping, or they were confined to datasets containing only TVS scans. Furthermore, many of these datasets are not publicly accessible, hindering reproducibility and further advancements in this field. This highlights the need for a more comprehensive and accessible dataset that includes both TVS and TAS modalities, aiming to eliminate the dependency on manual cropping and enhance the automation and efficiency of the segmentation process.

## 3 Materials and methods

### 3.1 Overview of proposed method

**Figure 1:**
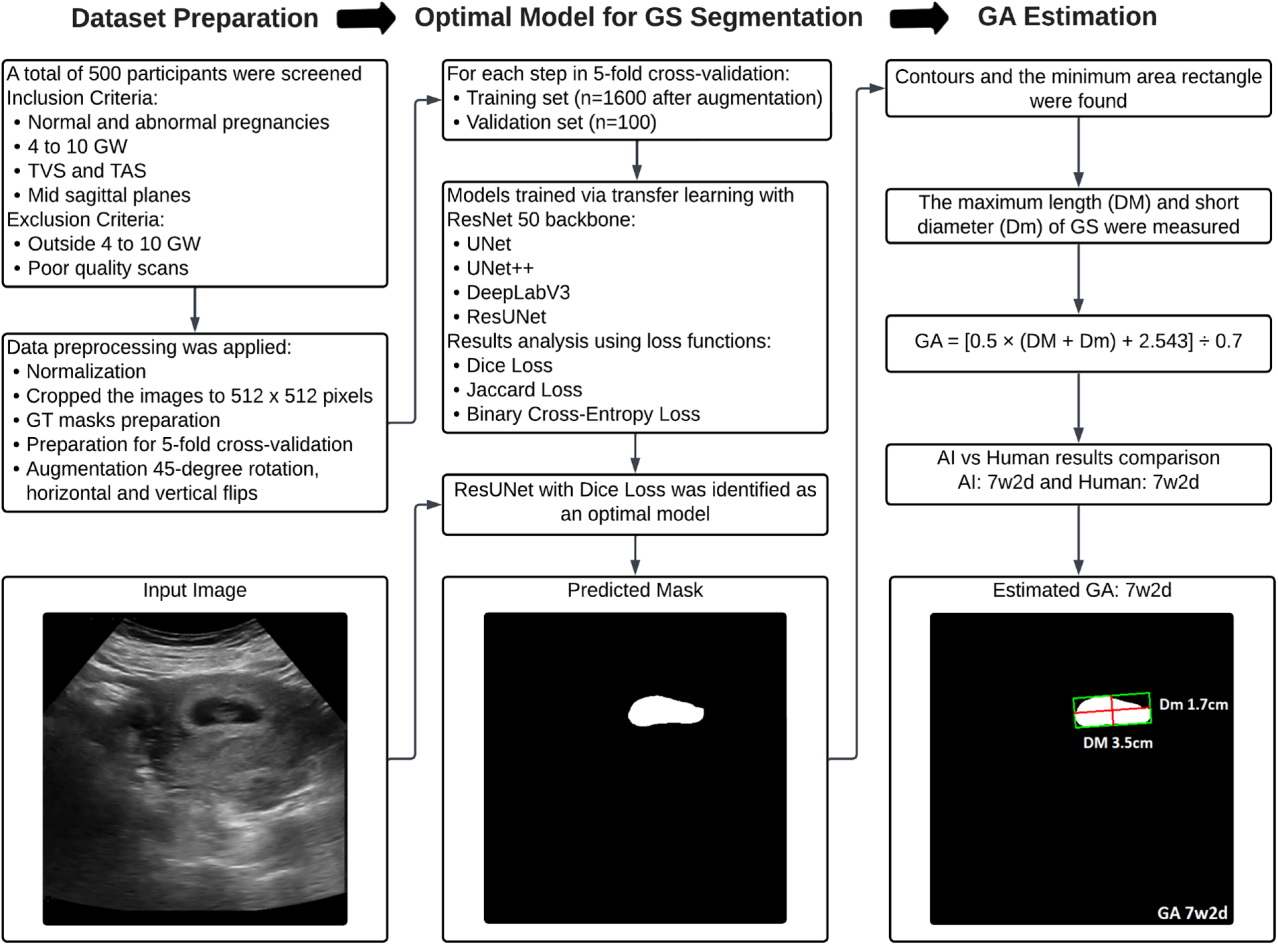
Flowchart illustrating the proposed methodology for dataset preparation, optimal model selection for GS segmentation, and subsequent GA estimation.

An overview of the proposed methodology for this research is presented in Figure 1. The pipeline is structured into three key stages to ensure a streamlined and systematic process for precise GS segmentation and accurate GA estimation. Step 1 involves image acquisition and dataset preparation, establishing a comprehensive dataset for training and validation. Step 2 focuses on selecting the optimal deep-learning model for GS segmentation. Finally, Step 3 implements an algorithm to estimate GA.

### 3.2 Image acquisition and dataset preparation

The study received approval from the Ethics Committee of The University of Lahore Teaching Hospital, Lahore, Pakistan, with registration number ERC/108/23/08. All experiments adhered to the principles outlined in the Declaration of Helsinki, and written consent was obtained from all participants before commencement. In this study, the US examination was conducted by using Canon Aplio 300, equipped with PVT-375BT (1.5–6.0 MHz) TAS transducer with center frequency 3.5 MHz and PVT-781VT (3–12 MHz) TVS transducer with center frequency 6.5 MHz. An accredited sonographer, Prof. Dr. Faiza Farooq, has 15 years of expertise and has conducted all scans. A standardized image acquisition protocol was adhered to, wherein the sagittal section of the uterus was depicted and the largest dimension of the GS was preserved. Parameters such as time gain compensation, uniform gain, signal-to-noise ratio, and dynamic range were configured in gynecological US mode.

**Figure 2:**
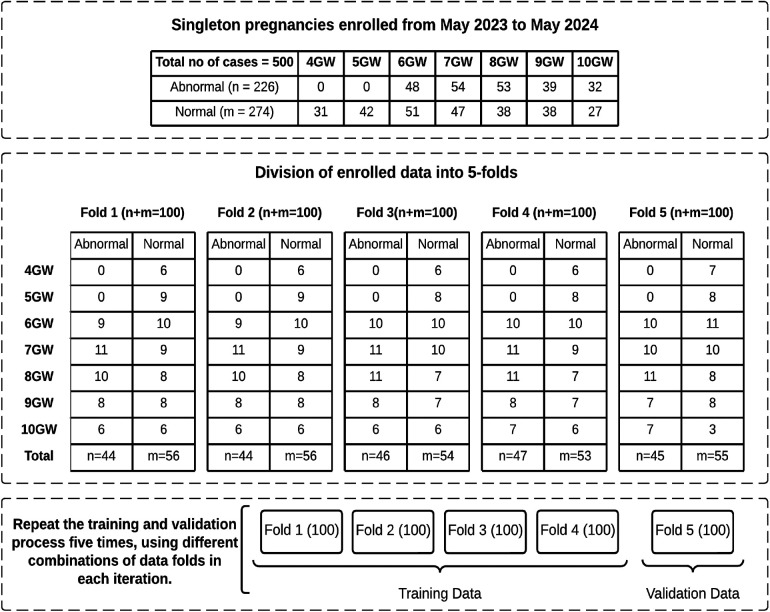
Overview of enrolled data with distribution of normal and abnormal cases across GW in each fold.

Five hundred US images of GS between 4–10 weeks gestation were stored in DICOM format. All images were presented in split-screen views, with one side displaying the original image and the other for biometric measurements. The split-screen images initially had dimensions of 720×960, which were subsequently cropped for anonymization purposes and to align with the segmentation network’s requirements, resulting in a final size of 512×512. The dataset includes 274 confirmed instances of normal fetal development, with 158 TAS and 116 TVS US images. Additionally, it contains 226 cases of miscarriage, encompassing scenarios like blighted ovum, absence of cardiac activity, irregular sac shape, and sudden pregnancy loss, with 147 TAS and 79 TVS US scans. Figure 2 provides a detailed overview of the data, including the distribution of abnormal and normal cases across GW.

Our dataset provides comprehensive pregnancy profiles for each patient, including details such as maternal age, body mass index (BMI), *in vitro* fertilization (IVF) or intracytoplasmic sperm injection (ICSI), history of recurrent pregnancy loss (RPL), and other relevant information, as summarized in Table 1. BMI measures body fat based on height and weight and is important to pregnancy health. Both high and low BMI levels are associated with pregnancy risks, including gestational diabetes, hypertension, and miscarriage. IVF is an assisted reproductive technology where an egg and sperm are combined outside the body in a laboratory, while in ICSI, a single sperm is directly injected into an egg to facilitate fertilization. By isolating pregnancies achieved through IVF or ICSI, researchers can better assess any unique risks or outcomes associated with this technique. RPL is defined as having two or more consecutive miscarriages, often indicating an underlying health issue in either partner. Documenting RPL in a patient’s profile is essential in miscarriage studies, as it allows researchers to identify factors that may elevate the risk of future pregnancy loss.

**Table 1:** Pregnancy profiles of women included in the study.

Parameter	Abnormal (n=226)	Normal (m=274)
Maternal age in years^a^	32 (22–46)	29 (19–40)
GA in weeks^a^	8 (6–10)	7 (4–10)
BMI^a^	25 (14–40)	24 (13–40)
Gravidity^a^	3 (1–5)	2 (1–4)
History of RPL^b^	6 (2.7)	17 (6.2)
IVF or ICSI^b^	27 (11.9)	15 (5.4)
Diabetes^b^	39 (17.3)	12 (4.3)
Hypertension^b^	57 (25.2)	38 (13.8)
Thyroid disorders^b^	23 (10.1)	17 (6.2)
Genetic disorders^b^	17 (7.5)	5 (1.8)
Smoking or drugs^b^	3 (1.3)	1 (0.4)

IVF (in-vitro fertilization), ICSI (intracytoplasmic sperm injection), RPL (recurrent pregnancy loss), BMI (body mass index).^a^Data is given as median (range).^b^Data is given as number (percent).

To establish the ground truth (GT) for GS segmentation, three experienced sonographers, each with over five years of expertise, voluntarily participated in the study. The process commenced with de-identified US scans to protect patient privacy, with each image assigned a unique identifier to ensure confidentiality. A training session was conducted to familiarize the sonographers with the GIMP 2.10.34 annotation tool and to standardize the marking procedure. Each sonographer independently delineated the GS region without knowing the others’ annotations. This process resulted in three distinct annotations per image. The final GT was then established by calculating the intersection of the GS regions annotated by all three sonographers, ensuring a consensus-based and highly accurate GT representation.

### 3.3 Segmentation networks

In this study, image segmentation was conducted using four fully convolutional neural networks: UNet, UNet++, DeepLabV3, and ResUNet. These architectures are extensively utilized in medical imaging research and are known for their reliability and efficiency (18). We employed transfer learning and fine-tuning to address the challenges of limited data availability and improve performance. Specifically, the original architectures were modified by replacing their encoders with a ResNet50 backbone pre-trained on the ImageNet dataset (19, 20). A brief description of each architecture is provided below.

#### 3.3.1 UNet

The classic UNet model was originally introduced for semantic segmentation by Ronneberger et al. (21). It is a leading medical image segmentation model used for various medical problems, with a strategy that heavily relies on data augmentation techniques to maximize the use of limited data. It comprises two main pathways: the encoder, which is responsible for capturing contextual information through compact feature maps, and the decoder, which facilitates precise localization via transposed convolutions. The encoder consists of multiple contraction blocks following a ConvNet-like architecture, employing a series of repeated two sets of 3×3 convolutions with ReLU and the max-pooling layer of size 2×2 with a stride of 2 to achieve downsampling. The expansive pathway encompasses the upsampling of feature maps along with 2×2 convolution (termed ’up-convolution’) to reduce feature channel dimensions, followed by concatenation with corresponding feature maps from skip connections. This is succeeded by two sets of 3×3 convolutions with ReLU. Ultimately, a 1×1 convolutional layer is employed to map the component feature vectors.

#### 3.3.2 UNet++

The UNet++ was developed by Zhou et al. (22) a modified version of UNet. It integrates three notable enhancements: deep supervision, dense skip connections, and redesigned skip pathways. The incorporation of redesigned skip pathways enhances the flow of information across various layers, facilitating a more effective fusion of features and preservation of contextual information. Meanwhile, dense skip connections establish direct connections between all layers in the network, enabling a seamless flow of both information and gradients throughout the architecture. Additionally, deep supervision integrates extra supervision signals at multiple intermediate layers, which aids in better gradient propagation during training, leading to improved learning of hierarchical features.

#### 3.3.3 DeepLabV3

The DeepLabV3 architecture is presented by Chen et al. (23). Atrous convolutions are extensively utilized throughout the network to capture multi-scale contextual information without significantly increasing the computational cost. The Atrous Spatial Pyramid Pooling (ASPP) module further enhances the network’s ability to capture context at various spatial resolutions by incorporating parallel atrous convolutional layers with different dilation rates. The decoder module refines segmentation results by upsampling feature maps and incorporating skip connections to recover spatial information lost during downsampling.

#### 3.3.4 ResUNet

**Figure 3:**
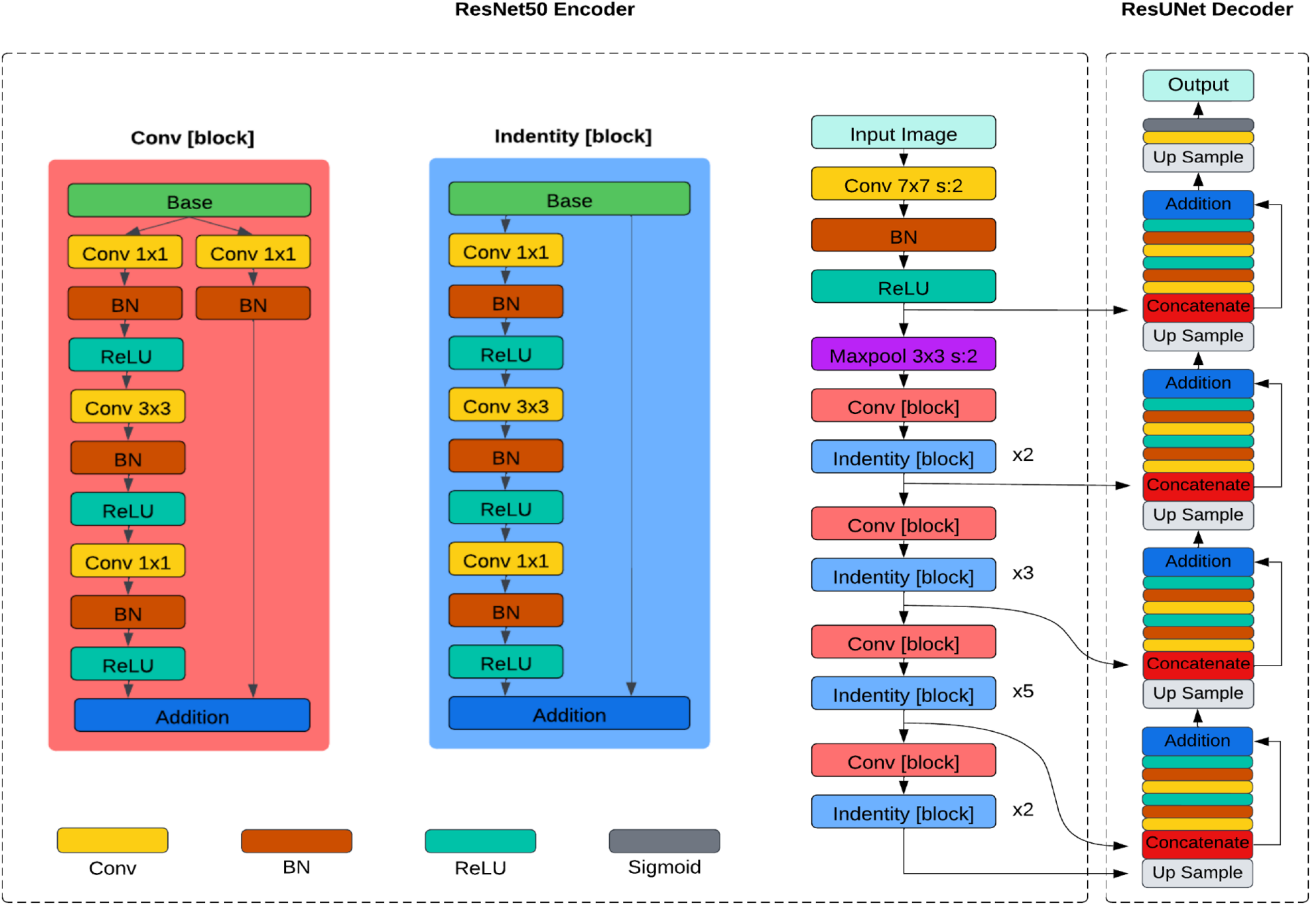
Architecture diagram of the ResUNet model using ResNet50 as the encoder.

The ResUNet, introduced by Zhang et al. (24), replaces the traditional encoder of U-Net with a pre-trained ResNet50 backbone in its architecture as depicted in Figure 3. The ResNet50 backbone comprises several residual blocks, each containing multiple convolutional layers. Similar to UNet, it has a contracting path (encoder) and an expansive path (decoder). The encoder downsamples the feature maps, capturing hierarchical features through convolution and pooling operations, and the decoder upsamples the feature maps to generate segmentation masks, using transposed convolutions or upsampling layers. Skip connections between corresponding layers in the encoder and decoder allow the model to capture local and global features while maintaining spatial information.

### 3.4 Loss functions

Loss functions measure how well the model performs on the training data (25). During the training process, minimizing the loss function enables the model to improve its predictive capabilities. Choosing an appropriate loss function involves considering various factors such as data characteristics, desired outcomes, and model attributes. DL (26, 27) and JL (28, 29) are selected for their capability to measure perfect overlap between predicted and GT masks, prioritizing boundary accuracy and aiding in precise delineation of structures. Conversely, BCEL (30, 31) is adept for binary segmentation tasks, effectively penalizing misclassifications and quantifying the minimal difference between predicted and GT masks, which contributes to faster convergence during training compared to other loss functions. Detailed explanations for each are provided below.

#### 3.4.1 Dice loss (DL)

For semantic segmentation, the DL is commonly used as a loss function to quantify the disparity between GT masks and predicted segmentation masks, as defined in Equation 1.

(1) $$\text{DL}=1-\frac{2|O_{\mathrm{GT}}\cap O_{\mathrm{PR}}|}{\left|O_{\mathrm{GT}}|+|O_{\mathrm{PR}}\right|}$$

Here, $O_{\mathrm{GT}}$ represents the GT mask, and $O_{\mathrm{PR}}$ is the predicted segmentation mask. DL encourages the model to produce segmentation masks with a higher overlap with the GT masks, resulting in more accurate segmentation.

#### 3.4.2 Jaccard loss (JL)

JL is utilized as a loss function in semantic segmentation tasks to measure the dissimilarity between $O_{\mathrm{GT}}$ and $O_{\mathrm{PR}}$. It is based on the Jaccard similarity coefficient, which calculates the intersection over the union of $O_{\mathrm{GT}}$ and $O_{\mathrm{PR}}$, as defined in Equation 2.

(2) $$\text{JL}=1-\frac{\left|O_{\mathrm{GT}}\cap O_{\mathrm{PR}}\right|}{\left|O_{\mathrm{GT}}\cup O_{\mathrm{PR}}\right|}$$

#### 3.4.3 Binary cross-entropy loss (BCEL)

BCEL can also be used for semantic segmentation tasks, especially when each pixel is treated as an independent binary classification problem. This loss function penalizes the model by assessing the difference between predicted probabilities and GT labels for each pixel independently, as defined in Equation 3.

(3) $$\text{BCEL}=-\frac{1}{N}\sum_{i=1}^{H}\sum_{\;j=1}^{W}[y_{ij}\log(p_{ij})+(1-y_{ij})\log(1-p_{ij})]$$

In this context, N represents the total number of pixels in the image, while H is the height and W is the image’s width. Here, $y_{ij}$ corresponds to the GT label of the pixel, where it is assigned 0 for the background and 1 for the foreground. Similarly, $p_{ij}$ signifies the predicted probability of the positive class for the pixel at position (i,j).

### 3.5 Training procedure and optimized parameters

In the first iteration of the 5-fold cross-validation (32, 33), we selected 4 folds (400 US images) for the training set and 1 fold (100 US images) for the validation set. The training set was then augmented by applying 45-degree rotations, along with horizontal and vertical flips, resulting in a total of 1,600 images. All models were trained using the PyTorch framework on an NVIDIA Tesla K80 GPU with 12GB of VRAM. The Adam optimizer was employed in all experiments due to its superior performance compared to other algorithms. A learning rate of 0.0001 was chosen, and a batch size of 2 was used for both the training and validation datasets to ensure efficient training. Table 2 shows the number of trainable parameters and the optimal number of epochs, determined by stopping the training when validation accuracy declined, as recommended by (34). These hyperparameters were optimized by monitoring the validation set performance. Additionally, to identify the optimal GS segmentation model for our dataset, we conducted extensive experiments by training our models using three different loss functions: DL, JL, and BCEL. In each subsequent iteration, we used 4 different folds for the training set and 1 fold for the testing set. All models were trained using the previously optimized hyperparameter settings and evaluated on the testing set. This process of training and testing was repeated 5 times.

**Table 2:** Total number of trainable parameters and epochs.

Models	Parameters	Epochs	
UNet	32521250	DL	60
		JL	60
		BCEL	65
UNet++	48985890	DL	55
		JL	55
		BCEL	55
DeepLabV3	39633986	DL	60
		JL	65
		BCEL	65
ResUNet	33435410	DL	50
		JL	55
		BCEL	50

### 3.6 Gestational age estimation

The binary predicted mask obtained from the segmentation model was input into our novel Algorithm 1 to estimate GW. A step-by-step explanation is provided below:

1. Read the segmented GS binary image.
2. Apply the Moore Neighbor Tracing boundary-following algorithm (35) to extract the contour of the binary image. This algorithm traces the outer boundary of connected components using the Moore neighborhood, which considers the eight surrounding pixels for connectivity.
3. Determine the minimum area rectangle of the GS contour. This rectangle has the smallest possible area among all enclosing rectangles and is oriented such that its sides are not necessarily aligned with the coordinate axes.
4. Identify the four corner points (c1, c2, c3, and c4) of the minimum area rectangle.
5. Extract the column pixel spacing ($PS_{col}$) and row pixel spacing ($PS_{row}$) from the metadata.
6. Calculate the maximum length (DM) and short diameter (Dm) of the minimum area rectangle using the modified Euclidean distance equations, as defined in Equations 4 and 5. Both DM and Dm are measured in centimeters (cm).
7. Compute the GW using the Hellman method (36), as defined in Equation 6.
  (4) $$\begin{array}{rl} & DM=\\ & \sqrt{(c2_{x}\times PS_{col}-c1_{x}\times PS_{col}{)}^{2}+(c2_{y}\times PS_{row}-c1_{y}\times PS_{row}{)}^{2}} \end{array}$$
  (5) $$\begin{array}{rl} & Dm=\\ & \sqrt{(c3_{x}\times PS_{col}-c1_{x}\times PS_{col}{)}^{2}+(c3_{y}\times PS_{row}-c1_{y}\times PS_{row}{)}^{2}} \end{array}$$
  (6) $$GW=\frac{0.5\times (DM+Dm)+2.543}{0.7}$$

**Algorithm 1:** Gestational age (GA) estimation algorithm.

1: **procedure** MainProcedure(BinaryImage, PixelSpacing)
2: **Input:** BinaryImage and PixelSpacing
3: **Output:** Gestational Age in weeks (GW)
4: contour = MooreNeighborTracing(BinaryImage,(1,1))
5: minAreaRactangle = MinAreaRectangle(contour)
6: Find corners c1,c2,c3,c4 form minAreaRactangle
7: $PS_{col}$ = PixelSpacing[0]
8: $PS_{row}$ = PixelSpacing[1]
9: dx1 = $c2_{x}\times PS_{col}-c1_{x}\times PS_{col}$
10: dy1 = $c2_{y}\times PS_{row}-c1_{y}\times PS_{row}$
11: dx2 = $c3_{x}\times PS_{col}-c1_{x}\times PS_{col}$
12: dy2 = $c3_{y}\times PS_{row}-c1_{y}\times PS_{row}$
13: DM = $\sqrt{(dx1{)}^{2}+(dy1{)}^{2}}$
14: Dm = $\sqrt{(dx2{)}^{2}+(dy2{)}^{2}}$
15: GW = [0.5×(DM+Dm)+2.543]÷0.7
16: **return** GW
17: **procedure** MooreNeighborTracing(I, $p_{0}$)
18: Define Moore 8-neighborhood)
19: Get the dimensions of the image I (rows, cols)
20: Initialize the boundary list B with the starting pixel $p_{0}$
21: Set p = $p_{0}$
22: Find pixel $p_{1}$ in the clockwise direction from $p_{0}$
23: Set $p_{0}$ as the current point p and $p_{1}$ as the current neighbor c
24: **repeat**
25: Let n be the current neighbor c
26: **for** each neighbor of p in the counterclockwise direction from n **do**
27: **If** (I[n[0],n[1]] == 1)
28: Set n as the current neighbor
29: Set p = n
30: Add p to B
31: Set c to the current neighbor of p in the counterclockwise direction from n
32: **until** p equals $p_{0}$ and c equals $p_{1}$
33: **return** B
34: **procedure** MinAreaRectangle(P)
35: Compute the convex hull of the points in P
36: Initialize min_area to a large value
37: Initialize R with null dimensions
38: **for** each edge e in the convex hull **do**
39: Compute the angle θ between e and the x-axis
40: Rotate all points in P by −θ degrees
41: Compute the bounding box of the rotated points
42: Compute the area of the bounding box
43: **if** the area is smaller than min_area **then**
44: Update min_area to the new area
45: Update R with the dimensions and orientation of the bounding box
**return** R as the minimum area rectangle

### 3.7 Evaluation metrics

#### 3.7.1 Region-based metrics

Four popular region-based evaluation metrics (37) for segmentation algorithms have been used in this paper. The IoU is calculated as the ratio of the intersection area between $O_{\mathrm{GR}}$ and $O_{\mathrm{PR}}$ regions to the area of their union, as defined in Equation 7. Here, $O_{\mathrm{PR}}$ denotes the predicted result and $O_{\mathrm{GT}}$ is the expert’s provided GT.

(7) $$\text{IoU}=\frac{\left|O_{\mathrm{GT}}\cap O_{\mathrm{PR}}\right|}{\left|O_{\mathrm{GT}}\cup O_{\mathrm{PR}}\right|}$$

The Dice coefficient is another metric used to quantify the similarity between two sets. It is computed as twice the intersection area between the $O_{\mathrm{GR}}$ and $O_{\mathrm{PR}}$ regions to the sum of their areas, as defined in Equation 8. Its range lies between 0 and 1, where a higher value indicates a superior segmentation outcome.

(8) $$\text{Dice}=\frac{2|O_{\mathrm{GT}}\cap O_{\mathrm{PR}}|}{\left|O_{\mathrm{GT}}|+|O_{\mathrm{PR}}\right|}$$

Recall evaluates a model’s capacity to accurately detect positive instances, quantified as the ratio of the intersection area between $O_{\mathrm{GR}}$ and $O_{\mathrm{PR}}$ regions to the area of $O_{\mathrm{GR}}$ region, as expressed in Equation 9. A high recall value signifies proficient identification of positive instances with minimal missed positive instances.

(9) $$\text{Recall}=\frac{\left|O_{\mathrm{GT}}\cap O_{\mathrm{PR}}\right|}{\left|O_{\mathrm{GT}}\right|}$$

Precision gauges the accuracy of positive instance identifications relative to all instances labeled as positive by the model. It is computed as the ratio of the intersection area between $O_{\mathrm{GR}}$ and $O_{\mathrm{PR}}$ regions to the area of $O_{\mathrm{PR}}$ region, as expressed in Equation 10. A high precision value signifies minimal false detections by the model.


(10) $$\text{Precision}=\frac{\left|O_{\mathrm{GT}}\cap O_{\mathrm{PR}}\right|}{\left|O_{\mathrm{PR}}\right|}$$

#### 3.7.2 Bland–Altman plots

Bland–Altman plots (38) serve as a graphical tool for assessing the agreement between two distinct measurements. Particularly useful when comparing two techniques that measure the same entity. These plots represent the discrepancies between the measurements from the two methods on the y-axis, with the mean of the measurements depicted on the x-axis. They provide valuable insights into potential systematic biases between the methods, aiding in identifying consistent differences in measurements.

## 4 Results

### 4.1 Evaluation of GS segmentation models

**Figure 4:**
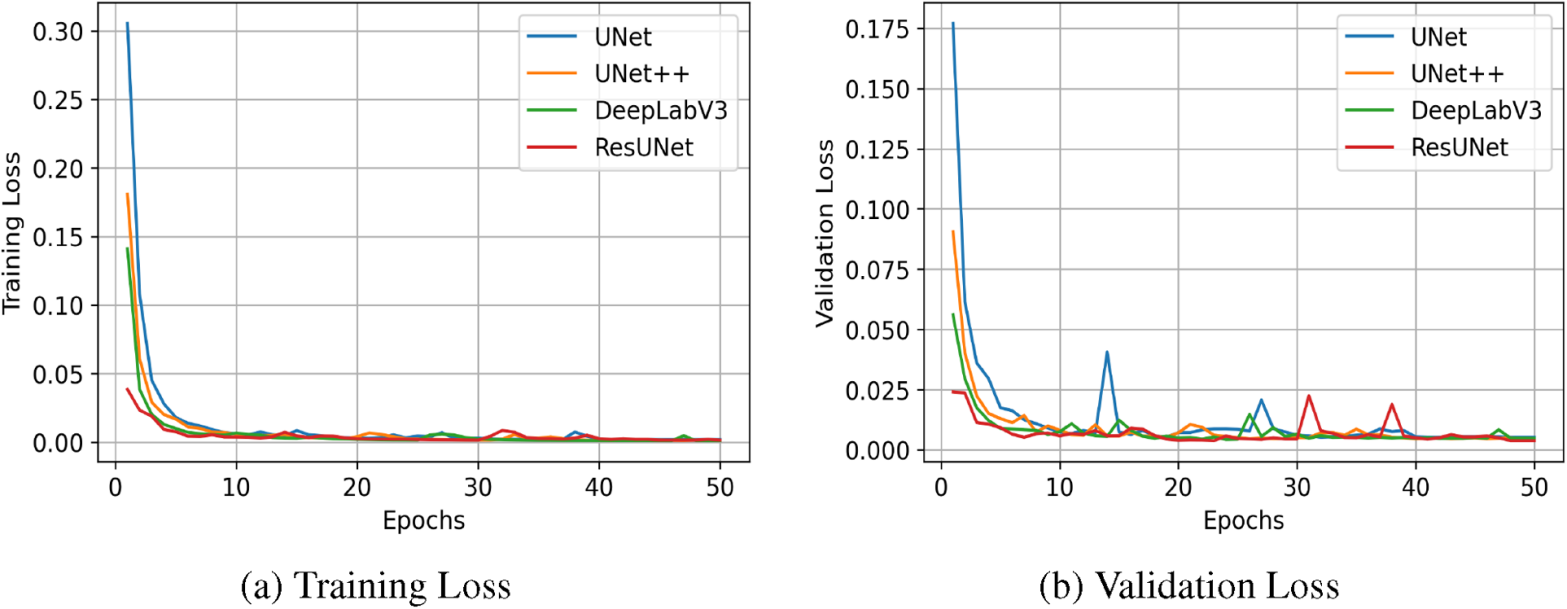
Learning curves measured by Dice Similarity Coefficient (DSC) for all compared segmentation models. **(a)** Training Loss, **(b)** Validation Loss.

**Figure 5:**
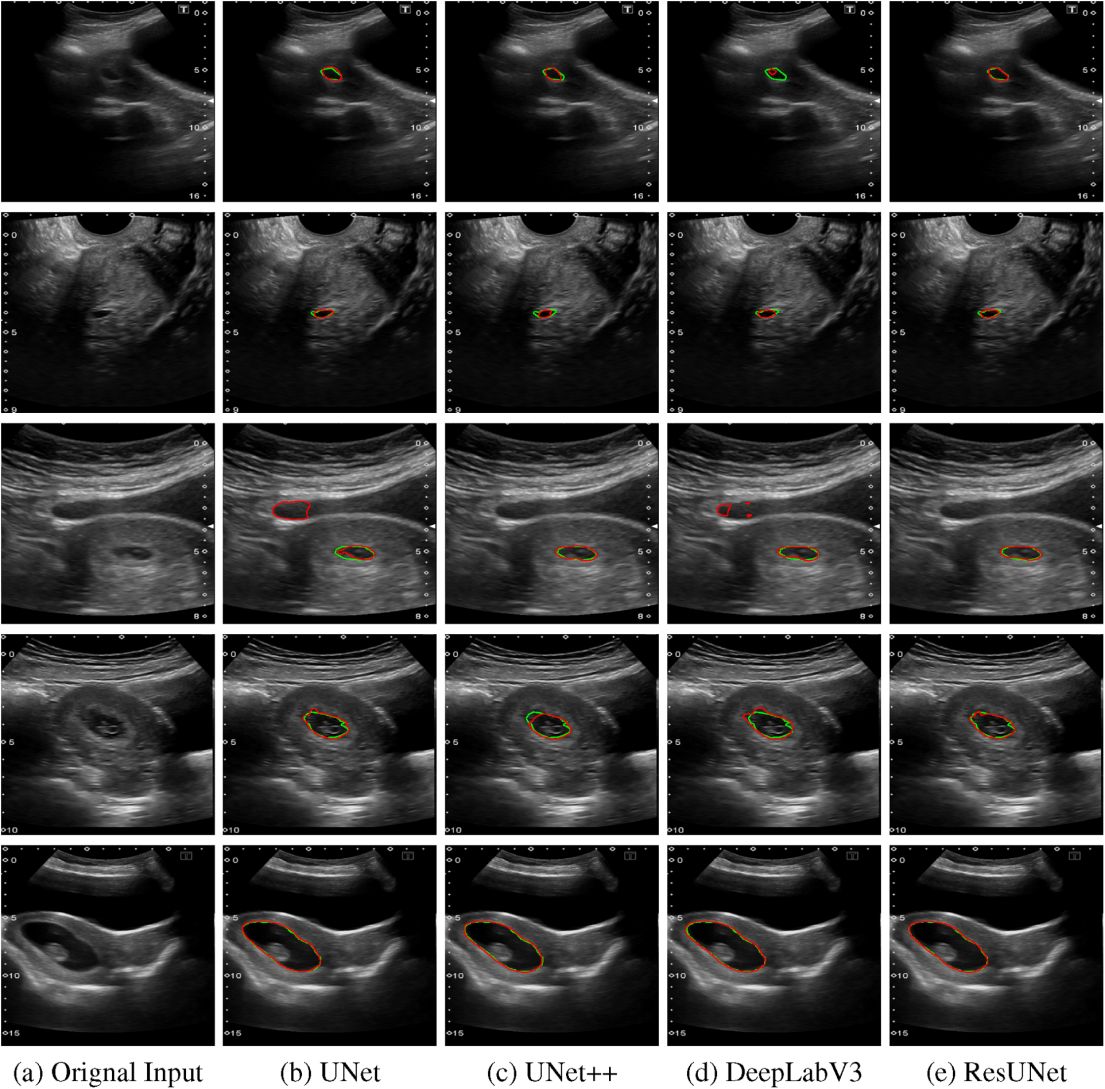
Comparison of segmentation results from various algorithms are presented: The first column shows the original US images, followed by segmentation outputs from UNet, UNet++, DeepLabV3, and ResUNet. Each row represents a distinct US image sample, with red contours indicating automated segmentation results and green contours representing the GT annotations. **(a)** Orignal Input, **(b)** UNet, **(c)** UNet++, **(d)** DeepLabV3, **(e)** ResUNet.

**Figure 6:**
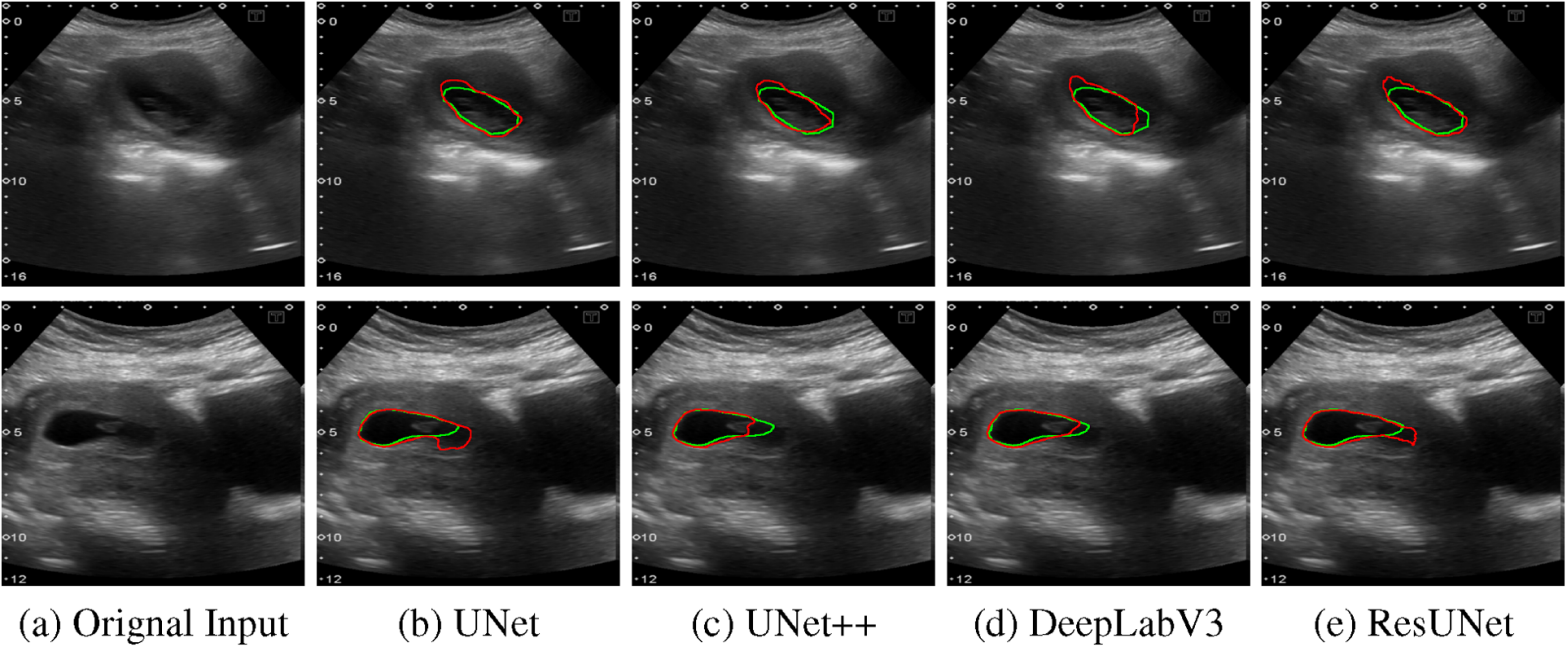
Two examples of poor segmentation results from various algorithms are presented: The first column displays the original US images, followed by segmentation outputs from UNet, UNet++, DeepLabV3, and ResUNet. Each row corresponds to a distinct US image sample, with red contours denoting automated segmentation results and green contours illustrating the GT annotations. **(a)** Orignal Input, **(b)** UNet, **(c)** UNet++, **(d)** DeepLabV3, **(e)** ResUNet.

The quantitative results for all segmentation models, each optimized with different loss functions in a single-fold validation, are presented in Table 3. Notably, ResUNet with DL achieved the highest performance, with an IoU of 92.73%, a Dice score of 96.2%, a Recall of 97.84%, and a Precision of 94.70%. Figure 4 illustrates the learning curves for all models, demonstrating smooth and stable trajectories that reflect successful training and convergence using the DL function. This configuration effectively balanced false negative (FN) and false positive (FP) detections, improving segmentation outcomes. Additionally, it demonstrated impressive processing efficiency, completing the task in just 1.5 s per image, as shown in Table 3. The qualitative comparisons of segmentation outputs are depicted in Figure 5, highlighting that ResUNet consistently achieves lower missed and false detection rates than the other methods. Additionally, two cases of poor segmentation, shown in Figure 6, are likely due to incomplete visibility of GS boundaries, which may have impacted the model’s performance in these instances.

**Table 3:** Evaluation of GS segmentation using different loss functions.

Models	Loss	IoU	Dice	Recall	Precision	Processing time(s)/image
UNet	DL	0.8647	0.9233	0.9525	0.9020	2.1
	JL	0.8592	0.9175	0.9283	0.9221	2.5
	BCEL	0.8577	0.9164	0.9374	0.9124	**1.5**
UNet++	DL	0.8660	0.9263	0.9539	0.9049	5.9
	JL	0.8449	0.8976	0.9249	0.8753	6.3
	BCEL	0.8601	0.9203	0.9498	0.9009	6.1
DeepLabV3	DL	0.8496	0.9119	0.9473	0.8948	4.6
	JL	0.8528	0.9101	0.9381	0.8864	6.1
	BCEL	0.8551	0.9146	0.9210	0.9273	4.6
ResUNet	DL	**0.9273**	**0.9621**	**0.9784**	**0.9470**	**1.5**
	JL	0.9258	0.9613	0.9752	0.9484	2.7
	BCEL	0.9172	0.9564	0.9694	0.9446	1.6

Bold values highlight the highest performance scores across various segmentation models with different loss functions, based on the evaluation metrics.

**Figure 7:**
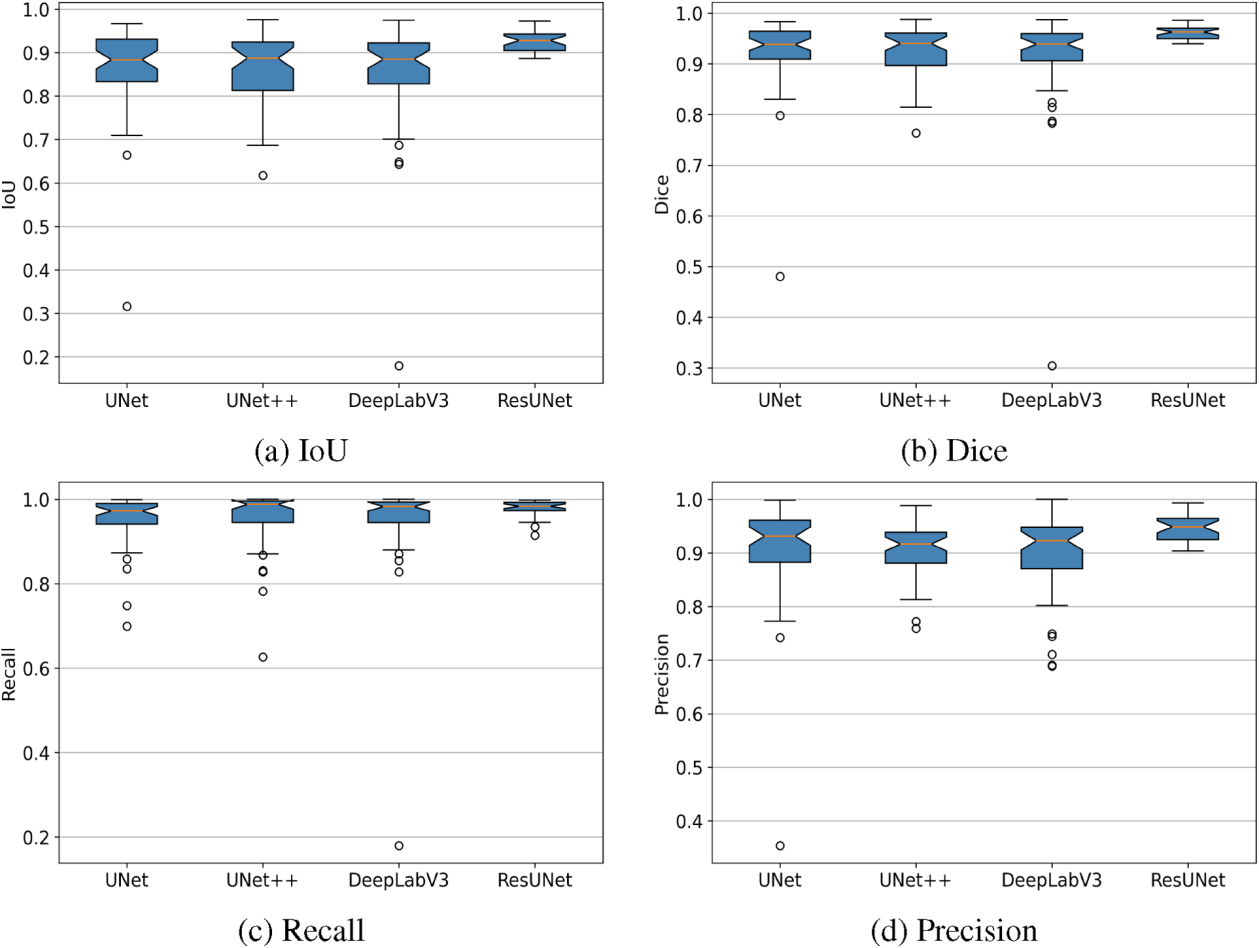
Box plots showing IoU, Dice, Recall, and Precision metrics achieved by different GS segmentation models. **(a)** IoU, **(b)** Dice, **(c)** Recall, **(d)** Precision.

**Figure 8:**
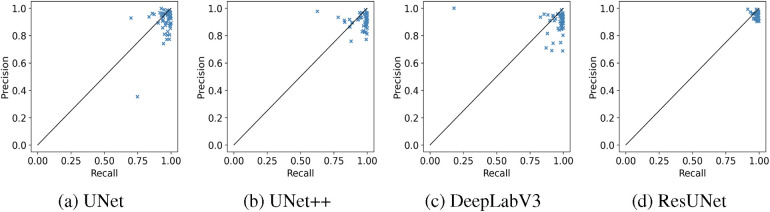
Recall-Precision analysis plots demonstrating the performance of different GS segmentation models. **(a)** UNet, **(b)** UNet++, **(c)** DeepLabV3, **(d)** ResUNet.

The box plots in Figure 7 demonstrate that ResUNet with DL achieved the highest average scores across all metrics, with the smallest fluctuation range, indicating greater stability. In contrast, other models exhibited lower average scores and wider fluctuation ranges, suggesting variability in segmentation quality. The correlation between Recall and Precision facilitates an in-depth analysis of segmentation performance. In Figures 8a–c, alternative segmentation methods demonstrate high Recall but low Precision, indicating a higher rate of false detections and a lower rate of missed detections. Conversely, Figure 8d highlights the performance of ResUNet with DL, which achieves an optimal balance between false and missed detection rates.

More reliable estimates of each model’s performance, obtained through 5-fold cross-validation, are presented in Table 4. ResUNet with DL achieved the highest mean scores across key metrics, with an IoU of 94.60%, a Dice coefficient of 97.80%, a Recall of 98.70%, and a Precision of 95.80%. This consistent performance across folds highlights ResUNet’s stability and makes it the optimal choice for achieving accurate and reliable GS segmentation in clinical applications. Table 5 demonstrates that our approach outperforms competing GS segmentation methods, achieving superior evaluation outcomes across all metrics.

**Table 4:** Evaluation of GS segmentation using 5-fold cross-validation (mean ± standard deviation).

Models	IoU	Dice	Recall	Precision
UNet	0.864±0.105	0.911±0.075	0.932±0.059	0.923±0.099
UNet++	0.873±0.076	0.925±0.046	0.941±0.070	0.909±0.052
DeepLabV3	0.854±0.125	0.914±0.100	0.927±0.118	0.910±0.074
ResUNet	0.946±0.017	0.978±0.009	0.987±0.008	0.958±0.017

The bold highlighted scores represent the highest performance of our method compared to state-of-the-art models and existing methods, based on the evaluation metrics.

**Table 5:** Comparison of segmentation results with competing methods (mean ± standard deviation).

Methods	IoU	Dice	Recall	Precision
Liu et al. (9)	0.785±0.051	0.855±0.049	0.866±0.027	0.884±0.030
Yin et al. (12)	0.585±0.032	0.690±0.046	0.637±0.021	0.859±0.075
Pei et al. (13)	0.852±0.037	0.909±0.034	0.917±0.026	0.902±0.030
This work	0.946±0.017	0.978±0.009	0.987±0.008	0.958±0.017

The bold highlighted scores represent the highest performance of our method compared to state-of-the-art models and existing methods, based on the evaluation metrics.

### 4.2 Evaluation of GA estimator

**Figure 9:**
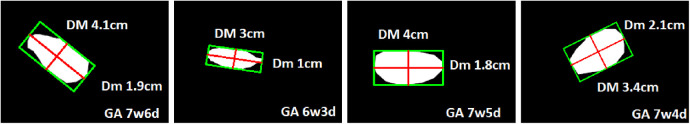
Examples of multi-angle GS measurements with corresponding GA estimations obtained using the proposed GA estimator.

**Figure 10:**
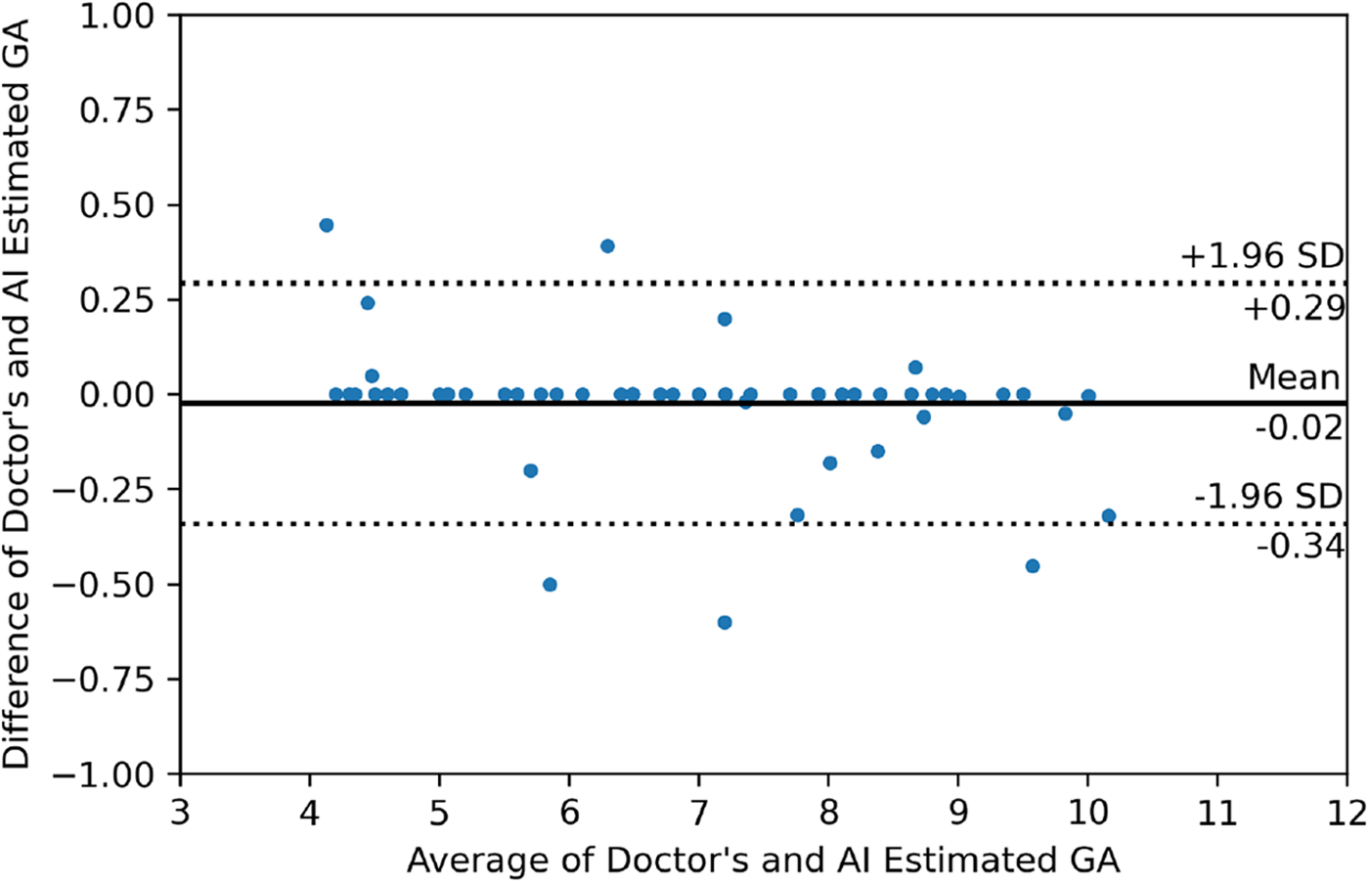
Bland–Altman plot illustrating the relationship between the differences and averages of GA estimates from the doctor and the AI-generated estimates.

Figure 9 shows the output of our GA estimation algorithm applied to various multi-angled GS examples. The algorithm accurately delineates a minimum-area rectangle around the segmented GS region, allowing for precise measurements of the DM and Dm. Additionally, Figure 10 presents the Bland–Altman analysis comparing GA estimates from our proposed pipeline with those provided by clinicians. The analysis reveals a mean difference of −0.02 weeks, indicating minimal bias between the two methods. The 95% limits of agreement were calculated at 0.29 and −0.34 weeks, with only five measurements falling outside this range, demonstrating a high level of agreement between the human (clinician) and machine (automated) estimates. Furthermore, the mean absolute error (MAE) was 0.07 weeks, further supporting the accuracy and reliability of our GA estimation pipeline.

## 5 Discussion

Accurate segmentation and biometric measurements of the GS are essential for monitoring fetal growth and development, identifying potential abnormalities, and improving predicting pregnancy outcomes. Traditionally, these tasks rely on clinicians’ manual measurements, which can be time-consuming and prone to variability, potentially impacting consistency and diagnostic accuracy. In this study, we developed an automated pipeline for GS segmentation and GA estimation to enhance the accuracy and consistency of diagnostic workflows. Four deep-learning models (UNet, UNet++, DeepLabV3, and ResUNet) were utilized for the GS segmentation task, and their results were analyzed and compared. ResUNet with DL function outperforms the other three by achieving the highest average scores across all metrics with the smallest fluctuation range. It exhibits greater stability and a balance between false and miss detection rates.

The Bland–Altman analysis allows researchers to visually evaluate the degree of agreement, identify systematic biases, and determine whether the machine’s performance aligns closely with that of clinicians. In our case, the analysis reveals that the GA estimator introduces minimal bias compared to clinician estimates, with the 95% limits of the agreement being narrow, indicating a high level of consistency between the two methods. This consistency is vital in clinical practice, where accurate and reliable GA estimation is crucial for patient care. The results suggest that our algorithm is well-suited for integration into clinical workflows, providing a more objective and consistent alternative to manual measurements.

Direct comparison with the previous studies was challenging due to the unavailability and variations in the datasets. Our dataset stands out in contrast to those used by Liu et al. (9), Yin et al. (12), and Pie et al. (13) due to its comprehensive inclusion of both TVS and TAS scans and its diverse representation of cases, covering both normal and abnormal pregnancies. Unlike the datasets used by (9, 13), which primarily consist of normal cases via TVS US examination, our dataset provides a broader perspective. Although Yin et al. (12) utilized both TVS and TAS scans, their collected samples were limited to 6 to 9 weeks of gestation, whereas our dataset spans a wider GA range from 4 to 10 weeks. Moreover, the techniques by (9, 12) require manual cropping or pre-processing to focus on the ROI. In contrast, our approach performs automatic GS segmentation, eliminating the need for manual ROI selection. This increased level of automation enhances workflow efficiency and makes our method more practical for real-time assistance in clinical settings. To enable a fair comparison, we re-implemented competing methods on our dataset, where our approach demonstrated superior performance.

The key contributions of this study include the development of a novel dataset comprising both TVS and TAS US scans, encompassing a diverse range of cases, including normal and abnormal pregnancies from 500 participants. We fine-tuned four widely utilized segmentation models, namely UNet, UNet++, DeepLabV3, and ResUNet, by integrating a pre-trained ResNet50 encoder, achieving state-of-the-art performance for each model. Our findings identified that using ResUNet with a ResNet50 encoder and DL provides an optimal solution for GS segmentation, representing a pioneering approach in this area. Furthermore, we developed a novel algorithm to perform biometric measurements of the segmented GS region, such as the DM and Dm, for accurate GA estimation. This advancement can assist clinicians by providing an accurate and consistent alternative to traditional manual methods for pregnancy assessment.

While our method yields promising results, there are some limitations to consider. First, this study was conducted as a single-center investigation, which may limit the generalizability of the findings across diverse clinical settings. Additionally, the sample size was also relatively small, which could restrict the model’s robustness and ability to generalize effectively across larger populations. Another limitation is the dependence on standard plane extraction, where the largest view of the GS is visible. These planes were manually identified and captured by experienced sonographers during live examinations, serving as the input images for the proposed pipeline. To address these limitations, future work will focus on expanding our dataset by collaborating with multiple centers. This collaborative approach will enable us to gather a larger and more diverse sample. Additionally, we aim to refine our pipeline by incorporating real-time image analysis, the system would identify the largest and most relevant GS view automatically, reducing reliance on manual selection and minimizing variability across sonographers. Moreover, we intend to broaden the scope of our research to include the segmentation and measurement of other fetal components.

## 6 Conclusion

In conclusion, we developed an advanced pipeline to support sonographers in accurately estimating GA. This study leveraged a novel dataset of early gestation US scans (4–10 weeks) from TVS and TAS modalities. Our evaluation of four segmentation models demonstrated that ResUNet with a ResNet50 encoder, optimized with DL, achieved superior performance in GS segmentation compared to existing methods. Additionally, we introduced a novel biometry-based approach for GA estimation, offering a robust and consistent tool for prenatal monitoring. This pipeline has significant potential to enhance accuracy, standardization, and efficiency in clinical settings.

## Written informed consent

Our study adhered to the ethical standards outlined in the 2008 Helsinki Declaration for all experiments involving human volunteers. We utilized an anonymized dataset, ensuring the confidentiality of all personal information. Additionally, informed written consent, which was prepared in both English and Urdu languages, is acquired from each volunteer during their initial hospital visit.

## Data Availability

The raw data supporting the conclusions of this article will be made available by the authors, without undue reservation.

## References

[1] Giakoumelou S Wheelhouse N Cuschieri K Entrican G Howie SE Horne AW. The role of infection in miscarriage. Hum Reprod Update. (2016) 22:116–33. 10.1093/humupd/dmv041 26386469 PMC4664130

[2] Wang Y Zhang Q Yin C Chen L Yang Z Jia S. Automated prediction of early spontaneous miscarriage based on the analyzing ultrasonographic gestational sac imaging by the convolutional neural network: a case-control and cohort study. BMC Pregnancy Childbirth. (2022) 22:621. 10.1186/s12884-022-04936-0 35932003 PMC9354356

[3] Farren J Mitchell-Jones N Verbakel JY Timmerman D Jalmbrant M Bourne T. The psychological impact of early pregnancy loss. Hum Reprod Update. (2018) 24:731–49. 10.1093/humupd/dmy025 30204882

[4] Lee W Deter R McNie B Powell M Balasubramaniam M Gonçalves L. Quantitative and morphological assessment of early gestational sacs using three-dimensional ultrasonography. Ultrasound Obst Gynecol Off J Int Soc Ultrasound Obst Gynecol. (2006) 28:255–60. 10.1002/uog.2840 16937412

[5] Brier N. Understanding and managing the emotional reactions to a miscarriage. Obst Gynecol. (1999) 93:151–5. 9916974 10.1016/s0029-7844(98)00294-4

[6] Tan S Tangal NG Kanat-Pektas M Özcan AŞ Keskin HL Akgündüz G. Abnormal sonographic appearances of the yolk sac: which can be associated with adverse perinatal outcome? Med Ultrason. (2014) 16:15–20. 10.11152/mu.2014.2066.161.st1gt2 24567919

[7] Condous G. Ultrasound diagnosis of miscarriage: new guidelines to prevent harm. Australas J Ultrasound Med. (2011) 14:2. 10.1002/j.2205-0140.2011.tb00127.x 28191122 PMC5024905

[8] Centre NC. Ectopic Pregnancy and Miscarriage: Diagnosis and Initial Management in Early Pregnancy of Ectopic Pregnancy and Miscarriage. London: RCOG Press (2012). 23638497

[9] Liu L Tang D Li X Ouyang Y. Automatic fetal ultrasound image segmentation of first trimester for measuring biometric parameters based on deep learning. Multimed Tools Appl. (2023) 00:1–22. 10.1007/s11042-023-16565-6

[10] Pinto A Pinto F Faggian A Rubini G Caranci F Macarini L. Sources of error in emergency ultrasonography. Crit Ultrasound J. (2013) 5:1–5. 10.1186/2036-7902-5-S1-S1 23902656 PMC3711733

[11] Yu Z Tan EL Ni D Qin J Chen S Li S. A deep convolutional neural network-based framework for automatic fetal facial standard plane recognition. IEEE J Biomed Health Inform. (2017) 22:874–85. 10.1109/JBHI.2017.2705031 28534800

[12] Yin C Wang Y Zhang Q Han F Yuan Z Yao Y. An accurate segmentation framework for static ultrasound images of the gestational sac. J Med Biol Eng. (2022) 00:1–14. 10.1007/s40846-021-00674-4

[13] Pei Y Han J Wang H Liang H. Combining deep learning and intelligent biometry to extract ultrasound standard planes and assess early gestational weeks. Eur Radiol. (2023) 33:9390–400. 10.1007/s00330-023-09808-5 37392231

[14] Khazendar S Farren J Al-Assam H Sayasneh A Du H Bourne T. Automatic segmentation and classification of gestational sac based on mean sac diameter using medical ultrasound image. In: >Mobile Multimedia/Image Processing, Security, and Applications 2014. Vol. 9120. SPIE (2014). p. 92–8.

[15] Otsu N. A threshold selection method from gray-level histograms. Automatica. (1975) 11:23–7. 10.1109/TSMC.1979.4310076

[16] Zhang L Chen S Chin CT Wang T Li S. Intelligent scanning: automated standard plane selection and biometric measurement of early gestational sac in routine ultrasound examination. Med Phys. (2012) 39:5015–27. 10.1118/1.4736415 22894427

[17] Ibrahim DA Al-Assam H Du H Farren J Al-karawi D Bourne T. Automatic segmentation and measurements of gestational sac using static B-mode ultrasound images. In: Mobile Multimedia/Image Processing, Security, and Applications 2016. Vol. 9869. SPIE (2016). p. 73–85.

[18] Khan MZ Gajendran MK Lee Y Khan MA. Deep neural architectures for medical image semantic segmentation. IEEE Access. (2021) 9:83002–24. 10.1109/ACCESS.2021.3086530

[19] He K Zhang X Ren S Sun J. Deep residual learning for image recognition. In: Proceedings of the IEEE Conference on Computer Vision and Pattern Recognition (2016). p. 770–8.

[20] Deng J Dong W Socher R Li LJ Li K Fei-Fei L. Imagenet: a large-scale hierarchical image database. In: 2009 IEEE Conference on Computer Vision and Pattern Recognition. IEEE (2009). p. 248–55.

[21] Ronneberger O Fischer P Brox T. U-net: convolutional networks for biomedical image segmentation. In: Medical Image Computing and Computer-Assisted Intervention–MICCAI 2015: 18th International Conference, Munich, Germany, October 5–9, 2015, Proceedings, Part III 18. Springer (2015). p. 234–41.

[22] Zhou Z Rahman Siddiquee MM Tajbakhsh N Liang J.Unet++: a nested u-net architecture for medical image segmentation. In: Deep Learning in Medical Image Analysis and Multimodal Learning for Clinical Decision Support: 4th International Workshop, DLMIA 2018, and 8th International Workshop, ML-CDS 2018, Held in Conjunction with MICCAI 2018, Granada, Spain, September 20, 2018, Proceedings 4. Springer (2018). p. 3–11. 10.1007/978-3-030-00889-5_1 PMC7329239 32613207

[23] Chen LC Zhu Y Papandreou G Schroff F Adam H. Encoder–decoder with atrous separable convolution for semantic image segmentation. In: Proceedings of the European Conference on Computer Vision (ECCV) (2018). p. 801–18.

[24] Zhang Z Liu Q Wang Y. Road extraction by deep residual u-net. IEEE Geosci Remote Sens Lett. (2018) 15:749–53. 10.1109/LGRS.2018.2802944

[25] Wang Q Ma Y Zhao K Tian Y. A comprehensive survey of loss functions in machine learning. Ann Data Sci. (2020) 9:187–212. 10.1007/s40745-020-00253-5

[26] Milletari F Navab N Ahmadi SA. V-net: fully convolutional neural networks for volumetric medical image segmentation. In: 2016 Fourth International Conference on 3D Vision (3DV). IEEE (2016). p. 565–71.

[27] Sudre CH Li W Vercauteren T Ourselin S Jorge Cardoso M. Generalised dice overlap as a deep learning loss function for highly unbalanced segmentations. In: Deep Learning in Medical Image Analysis and Multimodal Learning for Clinical Decision Support: Third International Workshop, DLMIA 2017, and 7th International Workshop, ML-CDS 2017, Held in Conjunction with MICCAI 2017, Québec City, QC, Canada, September 14, Proceedings 3. Springer (2017). p. 240–8. 10.1007/978-3-319-67558-9_28 PMC7610921 34104926

[28] Yuan Y Chao M Lo YC. Automatic skin lesion segmentation using deep fully convolutional networks with Jaccard distance. IEEE Trans Med Imaging. (2017) 36:1876–86. 10.1109/TMI.2017.2695227 28436853

[29] Mohajerani S Saeedi P. Cloud and cloud shadow segmentation for remote sensing imagery via filtered Jaccard loss function and parametric augmentation. IEEE J Sel Top Appl Earth Observ Remote Sens. (2021) 14:4254–66. 10.1109/JSTARS.2021.3070786

[30] Yi-de M Qing L Zhi-Bai Q. Automated image segmentation using improved PCNN model based on cross-entropy. In: Proceedings of 2004 International Symposium on Intelligent Multimedia, Video and Speech Processing, 2004. IEEE (2004). p. 743–6.

[31] Jadon S. A survey of loss functions for semantic segmentation. In: 2020 IEEE Conference on Computational Intelligence in Bioinformatics and Computational Biology (CIBCB). IEEE (2020). p. 1–7.

[32] Stone M. Cross-validatory choice and assessment of statistical predictions. J R Stat Soc Ser B (Methodol). (1974) 36:111–33. 10.1111/j.2517-6161.1974.tb00994.x

[33] Mosteller F Tukey JW. Data analysis, including statistics. Handb Soc Psychol. (1968) 2:80–203.

[34] Bengio Y Goodfellow I Courville A. Deep Learning. Vol. 1. Cambridge, MA, USA: MIT Press (2017).

[35] Gonzalez RC. Digital Image Processing. Chennai: Pearson Education India (2009).

[36] Hellman LM Kobayashi M Fillisti L Lavenhar M. Growth and development of the human fetus prior to the twentieth week of gestation. Am J Obstet Gynecol. (1969) 103:789–98. 10.1016/0002-9378(69)90575-4 5773744

[37] Udupa JK LeBlanc VR Zhuge Y Imielinska C Schmidt H Currie LM. A framework for evaluating image segmentation algorithms. Comput Med Imaging Graph. (2006) 30:75–87. 10.1016/j.compmedimag.2005.12.001 16584976

[38] Mansournia MA Waters R Nazemipour M Bland M Altman DG. Bland–Altman methods for comparing methods of measurement and response to criticisms. Glob Epidemiol. (2021) 3:100045. 10.1016/j.gloepi.2020.100045 37635723 PMC10446118

